# Multidisciplinary Assessment to Personalize Length of Stay in Acute Decompensated Heart Failure (OPTIMA II ADHF)

**DOI:** 10.4021/jocmr1154w

**Published:** 2012-11-11

**Authors:** Frank Dusemund, Martin Steiner, Andre Vuilliomenet, Christian Muller, Rita Bossart, Katharina Regez, Ursula Schild, Antoinette Conca, Andreas Huber, Barbara Reutlinger, Beat Muller, Werner C. Albrich

**Affiliations:** a Medical University Department of the University of Basel, Kantonsspital Aarau, Aarau, Switzerland; b Department of Cardiology, Kantonsspital Aarau, Aarau, Switzerland; c Medical University Department of the University of Basel, University Hospital Basel, Basel, Switzerland; d Department of Nursing, Kantonsspital Aarau, Aarau, Switzerland; e Department of Laboratory Medicine, Kantonsspital Aarau, Aarau, Switzerland; f These authors contributed equally to this work

**Keywords:** Acute decompensated heart failure, Triage process, Biopsychosocial assessment, Length of stay, Hospital-associated disability

## Abstract

**Background:**

Acute decompensated heart failure (ADHF) causes a substantial burden for health care systems. Data to rationally define the need for hospitalization or the appropriate length of stay (LOS) is limited. Our aim was to personalize length of stay in patients admitted to hospital for acute decompensated heart failure.

**Methods:**

Consecutive patients with ADHF presenting to our emergency department were prospectively followed. We daily conducted a multidisciplinary risk assessment and compared proposed with actually observed triage decisions.

**Results:**

At presentation, all patients required hospitalization. Median LOS was 11 days including 1 day after reaching medical stability. In 42.7% of patients, hospitalization was prolonged after medical stability mainly for nursing and organizational reasons. Within 30 days of enrollment, 7 (9.3%) patients were rehospitalized, 3 of them for persisting or relapsing heart failure.

**Conclusions:**

There appears to be potential to shorten inhospital stay in patients with ADHF mainly by providing post discharge ambulatory nursing care in order to improve resource utilization and to diminish “hospitalization-associated disability”.

## Introduction

Acute decompensated heart failure (ADHF) is a leading cause of hospitalization in patients older than 65 years and has emerged as a major public health problem [1]. The most important part of the large costs associated with this disease is caused by hospitalizations [2]. There is surprisingly limited data about defining the need for hospitalization or the best time point for hospital discharge with respect to the competing goals of patient safety and cost reduction and of course the avoidance of disadvantages of unnecessary (long) hospitalizations (for example, nosocomial infections and worsening of frailty). A recent publication reported, that a fraction of approximately one third of older medical patients (> 70 years) suffer from persisting “hospitalization-associated disability”, highlighting the necessity of avoiding not indicated hospitalizations and to reduce the duration of hospitalization as far as possible [3].

In analogy to our previous observational study regarding lower respiratory tract infections (LRTI) [4] we herein expanded our observation to enroll patients with acute decompensated heart failure with the aim to develop a triage algorithm for ADHF to be tested in future intervention studies.

We aimed to develop an algorithm to allow stratification into the best fitting environment, respecting medical conditions and needs on the one hand and nursing, biopsychosocial and functional needs on the other hand, due to the fact that many medical patients primarily require nursing care and psychological assistance due to general frailty; ADHF may possibly only serve as the trigger for hospital admission, like shown for LRTI in our previous observation [4].

We aimed to evaluate, if the entity of ADHF is comparable to LRTI in regard to the above mentioned aspects and to assess the potential to avoid unnecessary hospitalizations on the one hand and to shorten inhospital stay on the other hand in these patients by using a multidisciplinary patient assessment.

We describe a prospective observational quality-control survey of our current triage process of patients with ADHF. We aimed to identify in a descriptive manner the proportions of patients who would best be cared for at different levels of care based on an interdisciplinary risk assessment using clinical and biopsychosocial and functional scores and patient preferences with the addition of the biomarker NT-proBNP [5].

## Methods

### Subjects and study design

This was a prospective observational quality control survey to evaluate the current triage practice for patients with ADHF at our tertiary care Medical University Department. Between December 2010 and May 2011, consecutive adults admitted to the emergency department (ED) with ADHF were prospectively monitored.

During the first hours of hospital presentation, the diagnosis of ADHF was established by the ED resident and ED attending physician. Consultation by a board-certified cardiologist was requested if there was diagnostic uncertainty in order to confirm the diagnosis and perform an echocardiogram. To be eligible for study inclusion, patients had to present with ADHF defined as acute dyspnea NYHA class III or IV and a NT-proBNP level of at least 300 pg/mL [6]. The diagnosis of ADHF was additionally based on typical symptoms and clinical findings supported by appropriate investigations such as electrocardiogram, chest x-ray, and echocardiography as recommended by current guidelines of the European Society of Cardiology [6]. The study team had no influence on diagnosis or medical treatment. Exclusion criteria were: 1). pulmonary embolism and 2). underlying acute coronary syndrom with high probability of percutaneous coronary intervention or bypass surgery within the next 36 hours.

Patients were monitored from admission to hospital discharge. Psychosocial and functional assessments were performed upon hospital admission (post acute care discharge score (PACD)), and during hospitalization (PACD day 3, “Selbst-Pflege-Index” (SPI) on days 2 ± 1, 5 ± 1, 8 ± 1, 11 ± 1 and within 2 days of discharge); medical assessment was performed daily during hospitalization to determine clinical stability and overruling criteria, if appropriate. Patients were interviewed after they had reached medical stability to assess their preference for site of care and feasibility of outpatient care at this time. All available patients underwent a follow-up phone interview 30 days after enrollment.

The local Institutional Review Board classified this study as observational quality surveillance and waived the need for patient informed consent.

### Risk assessment

#### Medical risk

**Figure 1:**
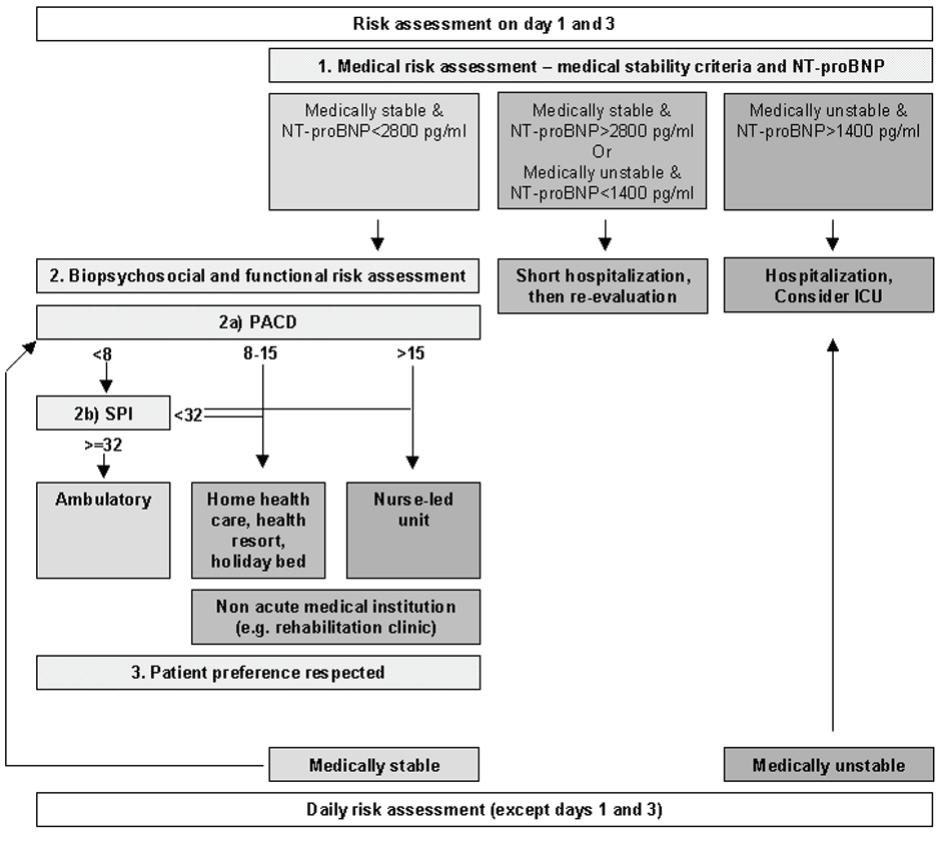
Virtual triage algorithm. PACD: post acute care discharge score; SPI: “Selbstpflegeindex”. At all time points of risk assessment, medical risk has been evaluated first. In case of medical stability, biopsychosocial and functional risk (“2.”) was determined: if then the PACD-score (“2a”) was below 8 points, SPI was calculated (“2b”). Then, the virtual preferred site of care (according to the arrows) was explained to the patient, who could deny beeing discharged.

At initial presentation in the ED, patients were classified into three medical risk categories (low risk, medium risk and high risk (Fig. 1)), depending on prespecified medical stability criteria. These were chosen according to the heart failure guideline of the Heart Failure Society of America [7], NT-proBNP levels [5, 8], and stability criteria derived from those for community-acquired pneumonia which had been used in our former observation [4, 9].

NT-proBNP cut-offs were chosen according to Logeart, where equivalent predischarge levels of BNP showed a hazard ratio of 5.1 or 15.2, respectively, for the risk of death or readmision [5]. The medical stability criteria are shown in Table 1. To be determined as medically stable, all medical stability criteria had to be fulfilled.

**Table 1:** Medical Stability Criteria

1. Marked reduction of most prominent admission sign (for example, dyspnea, edema, jugular venous distention, near complete resolution of rales)
2. Drop ≥ 30% of admission NT-proBNP
3. Stable oral medication and no i.v. therapy (diuretics, vasodilators, inotropics, vasopressors) for at least 24 h
4. Stable vital signs for at least 24 h (T < 37.8 °C, HR ≤ 100/min, RR ≤ 24/min, SO2 ≥ 90% or pO2 ≥ 60 mmHg on room air, SBP ≥ 90 mmHg)
5. Mental status back to baseline
6. No severe acute comorbidity necessitating hospitalization

T: temperature; HR: heart rate; RR: respiratory rate; SO2: O2-saturation; pO2: partial pressure of O2; SBP: systolic blood pressure.

#### Biopsychosocial and functional risk

The PACD score [10] was determined as a surrogate for biopsychosocial and functional status and nursing level requirement. Patients with a low medical risk were appropriate for care in non-acute medical institutions (for example, rehabilitation facility) and were further subgrouped into three risk categories according to PACD scores: we defined low biopsychosocial and functional risk (PACD < 8), appropriate for outpatient treatment; intermediate biopsychosocial and functional risk (PACD 8 - 15), appropriate for outpatient treatment with home-health aid, a holiday bed or stay in a health resort; or high biopsychosocial and functional risk (PACD > 15), appropriate for treatment in a proposed nurse-led unit (NLU). If the SPI-Index [11] was < 32 in patients with a low PACD score (< 8), the biopsychosocial and functional risk was considered intermediate.

The proposed triage algorithm combining medical, biopsychosocial and functional risk is shown in Fig. 1.

### Definitions

We prespecified optional overruling criteria which could be used by the treating physician to justify ongoing hospitalization despite formally fulfilling medical stability criteria. This overruling was virtual since some of these transfer options were not yet available for ADHF-patients, e.g. the NLU, or at times unavailable due to bed shortage.

Medical overruling criteria included: (1) acute illness requiring hospitalization independent from ADHF; (2) confusion, delirium or intravenous drug use.

Nursing and organizational overruling criteria were applicable when a patient was medically stable and allowed an increase in level of care up to the level of NLU: (1) SPI-Index < 32; (2) criteria requiring intensive nursing care, i.e. dementia, recurrent falls, decubitus ulcer and inability to reliably take medications; (3) waiting for non-acute medical care, i.e. holiday bed, rehabilitation, nursing home, home health care; (4) deficit of mobility or self-care requiring treatment; (5) other reasons, such as inconvenient timing (weekend, night).

Patients’ and relatives’ preferences were documented: (1) concern about safety at home; (2) lack of supporting social network; (3) other reasons.

### Endpoints

Our primary endpoint was to compare the percentage of patients allocated to the treatment locations based on the algorithm (Fig. 1) with the percentage of patients actually treated in these sites. Secondary endpoints were length of hospitalization before and after medical stability and identification of main reasons for discrepancy between actual and virtual treatment sites.

### Statistical analyses

Discrete variables were expressed as counts (percentage) and continuous variables as medians or means and standard deviations or interquartile range (IQR), unless stated otherwise.

## Results

### Baseline characteristics

Seventy-five patients were included in this survey (mean age 79.8 years; 57.3% male). The baseline characteristics are provided in Table 2.

**Table 2:** Baseline Characteristics


**Demographic characteristics**	(n = 75)

Mean Age (years)	79.8
Sex (male), no. (%)	43 (57.3)

**Coexisting illnesses, no. (%)**	

Cerebrovascular disease	5 (6.6)
Renal dysfunction	46 (61.3)
Pneumopathy	16 (21.3)
Malignancy	9 (12)
Diabetes	30 (40)
Peripheral artery disease	9 (12)
Any	71 (94.7)
Average count of coexisting illnesses	3.7

**Anamnestic findings, no. (%)**	

Orthopnea	49 (65.3)
Paroxysmal nocturnal dyspnea	36 (48)
Palpitations	15 (20)
Cough	34 (45.3)
Nocturia	31 (41.3)
Gain of weight	21 (28)
Angina pectoris	9 (12)
Limited exercise capacity	63 (84)
Average severity of dyspnea (NYHA)	3.4

**Clinical findings**	

Positive hepatojugular reflux (no./%)	49 (65.3)
Distended neck veins (no./%)	40 (53.3)
Lower extremity edema (no./%)	51 (68)
Rales (no./%)	53 (70.7)
Systolic blood pressure (mmHg)	129 (111 - 146)
Diastolic heart pressure (mmHg)	79 (68 - 89)
Heart rate (beats/min.)	92 (75 - 109)
Respiratory rate (breaths/min.)	20 (16 - 24)
Body temperature (°C)	36.7 (36.3 - 37.0)

**Diagnostic findings**	

LVEF, if echo performed (50/75 patients) (%)	44 (30 - 65)
Radiologic congestion (no./%)	43/74 (58.1)
NT-proBNP (ng/L)	14,154 (4,261 - 17,057)

No.: number; LVEF: left ventricular ejection fraction; data expressed as numbers and proportions or median and interquartil range unless stated otherwise.

### Allocation to treatment sites according to virtual triage algorithm

According to the proposed virtual triage algorithm for acute decompensated heart failure (Fig. 1), all 75 patients had a high medical risk at presentation in the emergency department, qualifying for hospitalization. Of these, 74 were hospitalized, whereas 1 patient left the ED against medical advice.

### Adverse events

In-hospital mortality was 8% (6/75), 30-day-mortality was 13.3% (10/75).

### Length of acute hospital stay

The median time of hospitalization was 11 (8 - 19) days.

### Time to medical stability

The median (IQR) time to medical stability, if achieved, was 10 (5.0 - 16.5) days, whereas 16 patients were discharged alive without ever having achieved medical stability according to our prespecified criteria.

We also evaluated length of stay after having reached medical stability, indicating the number of hospital days that could possibly be safely avoided. Overall, patients remained in hospital for a median of 1 (0 - 3) day after reaching medical stability.

### Overruling criteria

In 32 of all 75 patients (42.7%), hospital discharge was prolonged after stability criteria were fulfilled but overruled for the following reasons: medical overruling criteria in 9.4% of overruled cases, nursing and organizational overruling criteria in 62.5% and patients' preferences in 12.5%. In 15.6% of overruled cases, no specific reason was stated by the treating physician.

Acute other medical problems independent from acute decompensated heart failure was the only medical overruling criterion. The most frequent nursing or organizational overruling criterion was waiting for placement in a non-acute medical care facility. Patients' preferences would have been the primary reasons in 4 cases, due to concerns about safety at home (n = 2) or the lack of a supporting social network (n = 2) (Table 3). Interestingly, those patients, who were finally overruled for organizational or nursing reasons, already had a mean admission PACD score of 12.2.

**Table 3:** Reasons to Overrule Triage Algorithm After Medical Stabilization

Overruled cases total, no. (%)	32 (42.7)
Medical overruling criteria, no. (%)	3 (9.4)
Acute illness requiring hospitalization independent from CHF (no.)	3
Nursing and organizational overruling criteria, no. (%)	20 (62.5)
SPI-Index < 32 (no.)	3
Waiting for placement in a non-acute medical care facility (no.)	14
Other reasons (no.)	3
Patient's preferences, no. (%)	4 (12.5)
Concern about safety at home (no.)	2
Lack of supporting social network (no.)	2
No reason stated, no. (%)	5 (15.6)

No.: number; CHF: congestive heart failure, data expressed as numbers and proportions.

### Complications after discharge

Seven of seventy-five patients (9.3%) were rehospitalized within 30 days after initial presentation: 3 of these for persisting or relapsing heart failure, whereas 4 for different medical reasons independent from heart failure.

An unplanned consultation of the general physician within 30 days after initial presentation occurred in 2 of 75 cases (2.7%), one because of persisting heart failure and one because of a new gait disorder independent from heart failure.

## Discussion

The primary aim of this observational study was to assess how many patients might qualify for different treatment sites of a novel patient pathway using interdisciplinary, innovative triage criteria. We established an interdisciplinary recommendation for ideal treatment site by combining medical and biopsychosocial criteria, and considered patient’s preferences. This recommendation was, at this stage, still virtual.

Our first observation was that patients, who presented to the emergency department with acute decompensated heart failure were of old age (mean 80 years) and 94.7% of all patients suffered from at least one relevant comorbidity with a mean number of coexisting illnesses of 3.7. This indicates an even higher degree of overall morbidity in this cohort of patients in comparison to our former LRTI cohort (mean age 64 years, any comorbidity in 71.2%) [4].

According to the pre-defined medical stability criteria, all 75 patients were classified as medically unstable at initial presentation and therefore qualified for hospitalization. Patients remained medically unstable and consecutively in need of inhospital care for a median of 10 days. The actual median length of hospitalization was 11 days, indicating that patients stayed on average for one day longer than necessary from a medical point of view. This length of stay is similar to that of other studies investigating ADHF patients in Switzerland or other European countries [12-17], but longer than reported from ADHF cohorts in the USA [18-21]. Of note, our current cohort of patients showed a higher overall disease burden than reported from the US studies, which could at least in part explain a significantly longer length of stay. Bueno [19] documented a rehospitalization rate of 20.1% within 30 days with an increasing trend over the last years after a mean duration of inhospital stay of 7.6 days compared to a markedly lower rehospitalization rate of 9.3% within 30 days in our observation. This might indicate that patients in the USA might be discharged in less stable medical and psychosocial conditions due to different and stricter health policy requirements, resulting in significantly higher rehospitalization rates. It is unclear whether this leads to a lower overall financial burden of health care systems. Possibly the recent nation-wide implementation of DRG-based reimbursements might lead to a similar trend in Switzerland, underlining the urgent need to improve triage pathways for more efficient utilization of limited resources. Importantly, the canton of Aargau has used a DRG-based reimbursement system since 2004.

In the current observation, 32 of 75 patients (42.7%) were discharged later than medical stability was achieved, resulting in an overall median prolongation of inhospital stay of 1 day. Three of these were due to other acute medical problems independent from ADHF and therefore might be inevitable. In view of the observation that all admitted patients indeed required hospitalization based on our criteria, the potential for earlier discharge is the most important finding of this analysis.

The most frequent reasons (n = 20) for overruling our virtual triage pathway were nursing and organizational factors (low SPI-index: 3; waiting for placement in a non-acute medical care facility: 14; other reasons: 3). Therefore it seems most useful and appropriate to focus on reducing the waiting time for discharge to institutions like post acute care facilities (for example nurse-led units), nursing homes or old people’s homes, or increasing these capacities, respectively. The benefit of nurse-led clinics or the introduction of advanced practice nurses has been experienced in different countries and settings [22, 23] and is currently evaluated in an interventional study in LRTI patients in our institution. The fact that this group of 20 patients already initially had a PACD score of 12.2 in average reflects the good predictive value of initial PACD scores. It indicates that patients with higher PACD scores at entry could benefit from early efforts to establish a post-discharge plan.

In two cases, discharge was delayed because of the patients’ concerns about their safety at home, and another two patients reported the lack of a supporting social network. These factors could also be addressed by an improved ambulatory nursing supply. Also patient education before planned discharge and improved communication between hospital physicians and general practitioners to define a “post-discharge plan” could reduce fears and concerns of the patients [24].

The strength of this survey is its innovative and interdisciplinary concept including an individualized multifactorial risk assessment. We included all consecutive patients independent of severity of illness, cognitive status and comorbidities strengthening the generalizability to different settings and populations including the frail and cognitively impaired elderly, who otherwise are frequently excluded from randomized controlled trials.

The main limitation is the rather small sample size and thus insufficient power for further subanalyses. Since all patients required hospital admission, we were unable to draw further conclusions on our admission algorithm.

We defined stability criteria according to US guidelines [7] and published NT-proBNP levels [5, 8]. Parts of the recommended criteria are based on fairly subjective clinical signs and symptoms which may be judged differently in different populations and health-care settings by both healthcare providers and patients. NT-proBNP levels have been shown to correlate with rehospitalization and adverse outcomes in observational studies [5] but to our knowledge have not been tested to guide triage in intervention studies. It furthermore is unclear whether absolute values or kinetics may be preferable for this purpose. Since it was an observational design, our hypotheses should be tested in different settings in an interventional study. Obviously different capacities of outpatient management and follow-up greatly affect hospitalization rates and LOS.

In conclusion, the potential to avoid unnecessary hospitalizations or to shorten inhospital stay by improving triage processes in patients hospitalized for acute decompensated heart failure is lower than for patients with LRTIs from the same institution [4], due to a higher degree of morbidity and proportionally longer time of medical instability. Nevertheless, this potential should be utilized appropriately to avoid “hospitalization-associated disability” in view of limited healthcare resources.

### Contributors

FD, WA and BM had the idea, wrote the protocol and initiated the study. FD, WA, RB, KR, US and AC designed the questionnaire, managed the trial and collected data. FD performed the statistical analyses, FD drafted the manuscript, all other authors amended and commented on the manuscript. All authors approved the final version.

## Abbreviations

ADHF: acute decompensated heart failure
BNP: brain natriuretic peptide
DRG: disease related groups
ED: emergency department
IQR: interquartil range
LRTI: lower respiratory tract infections
NLU: nurse led unit
NT-proBNP: n-terminal precursor of brain natriuretic peptide
OPTIMA: optimized patient transfer using an innovative multidisciplinary assessment
PACD: post acute care discharge score
SPI: “Selbstpflegeindex”
